# Everyday Living with Diabetes Described by Family Members of Adult People with Type 1 Diabetes

**DOI:** 10.1155/2013/967872

**Published:** 2013-12-18

**Authors:** Tuula-Maria Rintala, Eija Paavilainen, Päivi Åstedt-Kurki

**Affiliations:** School of Health Sciences, Nursing Science, University of Tampere, Kuntokatu 4, 33520 Tampere, Finland

## Abstract

The aim of this study was to explore family members' experiences of everyday life in families with adult people living with type 1 diabetes. The grounded theory method was used to gather and analyse data from the interviews of nineteen family members. Six concepts describing the family members' views on everyday living with diabetes were generated on the basis of the data. Everyday life with diabetes is described as being intertwined with hypoglycemia. Becoming acquainted with diabetes takes place little by little. Being involved in the management and watching self-management from the sidelines are concepts describing family members' participation in the daily management of diabetes. The family members are also integrating diabetes into everyday life. Living on an emotional roller-coaster tells about the thoughts and feelings that family members experience. Family members of adult people with diabetes are involved in the management of the diabetes in many ways and experience many concerns. The family members' point of view is important to take into consideration when developing education for adults with diabetes.

## 1. Introduction

Diabetes mellitus is one of the most common chronic diseases all over the world. The aim of diabetes management is good metabolic control and the prevention of diabetes-related complications. Self-management has an important role in the management of diabetes [1, 2]. It is also recognised that family plays a significant part in the management of diabetes [3–8]. The importance of the relationship between the parents and the child with diabetes has been clearly documented [9–12]. In addition to that, parental support has been shown to enhance diabetes control and self-management in children and adolescents [9, 13]. During the adolescence, the collaboration with parents' decrease and the person with type 1 diabetes take responsibility of his/her self-management [14]. However, family members may provide practical help in the day-to-day management of diabetes also for adults with diabetes, as well as support and encouragement in lifelong adherence to a demanding regimen [4, 15, 16].

While family influences the self-management of diabetes, the diabetes of one family member also influences other members of the family. Parents of the children's with diabetes describe that their life is different after diabetes diagnosis and not so easier than before; they have lost their freedom. On one hand, they feel healthy and confident, and on the other hand the child is invisibly ill and they feel insecure and concerns connected to the future. Parents also have long-term emotional responses to the diabetes even several years after diagnosis [17–19].

Connected to the adult people with diabetes, it has been shown that family members perceive diabetes as a more serious illness than those suffering from diabetes themselves [20]. Family members experience different fears and worries connected with diabetes. They live with a constant concern for the health of the person with diabetes [21] and are uncertain of the future [22]. The concern about hypoglycemia is a diabetes-specific problem faced by couples living with type 1 diabetes. In Jørgensen et al. [23] study, family members even recall more episodes of severe hypoglycemia than the persons with diabetes. In addition to worries, family members experience lower positive well-being than the persons with diabetes [24]. Fisher et al. [25] found in their study that partners of persons living with type 2 diabetes experienced even higher levels of psychological distress than the persons with diabetes themselves. This was especially true with regard to female partners. Family members also seem to think that diabetes is a burdensome illness that one cannot control [26].

Maintaining a good metabolic balance on a daily basis is challenging from the family members' point of view as well [3]. In Armour et al.'s [9] systematic review about the effectiveness of family interventions, they found that people with diabetes had better metabolic control when a spouse was involved in an intervention. Family members want to be involved in the management of diabetes and support the person with diabetes [21], but they need knowledge about the treatment of diabetes and emotional and informational support for themselves as well [24, 27].

It is known that family relationships can provide an important source of support for the person with diabetes [9, 12, 16, 28]. It is also known that by causing fears and worries, the diabetes affects family members as well. The self-management of type 1 diabetes might be even more challenging than the self-management of type 2 diabetes due to the more demanding insulin treatment and self-monitoring of blood glucose, and the support from family members is crucial [29]. To find the best ways to support family members in their important and challenging role, it is vital to know what everyday life comprises and how the family members of adult people with type 1 diabetes experience their life. The aim of this study was to explore family members' experiences of everyday life in families with adult people with type 1 diabetes.

## 2. Method 

In this study the grounded theory method was used. It is a suitable method, when there is little knowledge about the phenomenon under study or a new perspective on the phenomena is required. The aim in grounded theory is to generate theory inductively through the process of constant comparison [30]. This study is part of a larger study where both the adult people with type 1 diabetes (*n* = 19) and their family members (*n* = 19) were interviewed. The participants with type 1 diabetes were obtained from one Diabetes Clinic and from one Diabetes Association in Finland. The eligibility criteria for the participants were that they had to be adults (aged over 18) and living with type 1 diabetes. The nurses of the Diabetes Clinic and the members of the Diabetes Association's board identified all potential participants and gave the study information letter and consent form to those who met the eligibility criteria and were willing to describe their everyday lives. The persons with diabetes nominated the family members to be included in this study. The participants were recruited until the theoretical saturation of the data was achieved [30].

### 2.1. Data Collection and Analysis

The data were collected by interviewing nineteen family members of adult people with type 1 diabetes. All interviews were conducted by the first author. The interviews took place in the participants' homes or in the first author's office. The duration of the interviews varied from 40 minutes to 2 hours. The family members were asked to share their experiences of everyday life with diabetes. Both the person with type 1 diabetes and the family members were present at the same time. During the first few interviews there were some preliminary questions for the interviews but later the themes in the interviews were based on what was discovered during the analysis of the previous interviews. With the participants' permission, the interviews were audio-recorded. All interviews were transcribed verbatim.

The analysis followed the basic steps of grounded theory, starting with an open coding of the data, then followed by axial and selective coding. First, the verbatim transcribed text (359 pages) was read. The open coding allowed creating 925 substantive codes. Then codes with similar contents were clustered into 26 subcategories (Table 1).

During the axial coding subcategories with similar properties were listed under a specific main category (see Table 2). Hence, concepts describing everyday living with diabetes were based on six main categories.

### 2.2. Ethical Considerations

The Ethics Committee of the Pirkanmaa Hospital District has given a favourable statement for the research plan of this study. Approval for this study was also obtained from the University Hospital and from the Diabetes Association. The participating adult persons with diabetes were provided with written information. The permission to audio-record the interview was also asked.

## 3. Results

Nineteen family members of adult people with type 1 diabetes were interviewed. Family members were spouses of the persons with diabetes, including one wife, 15 husbands, and three adult daughters of the people with diabetes.  Table 3 shows some background information of informants. All the other interviewed family members lived together with the person with diabetes, except one adult daughter. Only in one family the diabetes was diagnosed during marriage. In other families, the diabetes was diagnosed during the childhood or adolescence of the person with type 1 diabetes.

Six concepts describing family members' views on everyday living with diabetes were generated on the basis of the data: “intertwining with hypoglycemia,” “becoming acquainted with diabetes," “being involved in the management of diabetes," “integrating diabetes into everyday life," “watching self-management from the sidelines," and “living on an emotional roller-coaster."

### 3.1. Intertwining with Hypoglycemia

The danger of hypoglycemia is always present on the family members' minds. Over the years, they had learned to identify the signs of oncoming hypoglycemia. They recognised hypoglycemia based on very mild signs, such as the behaviour of the person with diabetes. They even noticed those signs earlier than the persons with diabetes themselves. Family members made various arrangements in order to avoid hypoglycemia, for example, planned their daily schedules carefully. They are also prepared for hypoglycemia in many different ways. They kept sweets with them all the time, in addition to having something edible all around the house, for example, sweets in the kitchen cabinets or jelly on the bedside table. Family members also offered help to the persons with diabetes during hypoglycemia. They urged the person with diabetes to perform blood glucose self-measurement, or they urged the person with diabetes to eat fast-acting carbohydrates. Family members even fed the persons with diabetes if they were not able to eat by themselves. The fear of severe hypoglycemia was common among the family members, especially with regard to those with earlier experiences of severe hypoglycemia.

*So, you have to remember the risk of hypoglycemia, always. Whatever you do, you must first pay attention to it and plan things well. *(family member 3B)**

*Then I started to pick up lumps of sugar from restaurants and kept them in my pockets all the time during my adolescence…  just in case mother would need help. *(family member 2B)**

*She started to act kind of nervously and speak quickly, so I said to her that you must take your blood glucose now… then I gave her juice. *(family member 15B).**

### 3.2. Becoming Acquainted with Diabetes

Becoming acquainted with diabetes is a lifelong process. At the beginning of their relationships, the spouses of the persons with diabetes had quite little information about diabetes. They experienced that they needed more information about the characteristics of diabetes and its treatment. Gathering information little by little from different sources and learning on their own were the methods the family members had used to become acquainted with the nature of the disease and the cornerstones of the treatment. The family members obtained most of the information about diabetes from the persons suffering from diabetes. Only a few of them had participated in any diabetes education in a health centre or hospital. In small doses, the persons with diabetes had told about the demands of the care, showed how the equipment needed to be used, and explained how to operate in acute situations.

*I learnt about the treatment of diabetes by following from the sidelines, watching my wife, and listening to what she tells. *(family member 5B).**

Over the years, diabetes had become a natural part of the family members' everyday life, and they had become used to it. They possessed a great deal of tacit knowledge, and they did not have to think about diabetes all the time anymore. Their conception of diabetes had changed and they perceived diabetes as a slight illness or no illness at all. Family members having long-time experience of diabetes could not imagine a life without it. They even considered diabetes as an individual property of the persons with diabetes.

*I do not consider diabetes an illness anymore—my wife has blue eyes, dark hair, and diabetes. *(family member 13B).**

### 3.3. Being Involved in the Management of Diabetes

The family members were interested in becoming involved in the management of diabetes. They wanted to better understand the self-management of diabetes in order to be more supportive to the persons with diabetes. They showed interest in the treatment equipment and its operation. The family members participated in the everyday self-management of diabetes by offering concrete help. They occasionally administered insulin injections to the persons with diabetes, and from time to time they performed blood glucose measurements on behalf of the persons with diabetes. They picked up treatment equipment on behalf of the persons with diabetes and reminded them of blood glucose measurements, eating, or exercising. The family members shared the healthy diet with the persons with diabetes—it is not necessary to prepare different food for the other members of the family.

*I am the reminder for my wife… remember to take your insulin with you, remember to eat, and remember to make self-measurement of blood glucose… and so on. *(family member 18B).**

The discussion about the diabetes regimen is another way to be involved in the management of diabetes. For family members, it was easy to talk about all aspects of diabetes and its management on the daily basis. One common topic for conversation was the daily condition of the persons with diabetes and the blood glucose level at that moment. On the other hand family members only talked about diabetes in connection with practical things, for example, whether injecting the insulin had been painful. Discussion about diabetes was even avoided.

*Then we discuss what the blood glucose value was and how to titrate insulin doses. *(family member 14B)**

*Diabetes is a taboo; we do not utter that word aloud in our family. *(family member 1B).**

Living in a constant awareness of the condition of the person with diabetes was mentioned as one way to be involved. The family members checked the condition of the persons with diabetes every once in a while. They ensured that everything was in order by calling the persons with diabetes when being away. Alongside their everyday activities, they kept an eye on the persons with diabetes all the time.

### 3.4. Integrating Diabetes into Everyday Life

The family members felt diabetes to be overwhelming and difficult to handle, and they experienced that it restricted their daily life. Planning the day-to-day living especially carefully is necessary for meeting the daily demands of diabetes and its treatment. The time-action profile of insulin must be taken into consideration, and everyday activities must often be carefully planned, for example, times for meals and exercise. In addition to that, the family members must live according to the schedule without any exceptions.

*Everyday life is scheduled; you must plan when you eat, when you exercise—you must plan your every movement. *(family member 13B).**

Family members described diabetes as a burdensome illness which affects everything they do. Diabetes was always on their minds, and they had to take diabetes into consideration every moment. They felt the loss of spontaneity caused by the scheduled treatment of diabetes. Spouses described sleep disturbances and said they awoke by various sounds supposing the person with diabetes had hypoglycemia. The family members hoped they could leave diabetes behind, but they considered it impossible. On the other hand, they thought that their lives were quite usual. Diabetes did not restrict their lives too much and they felt it was possible for them to do everything they wanted.

In families with little children, the children's games were related to diabetes as well. The children imitated injecting insulin and described having the symptoms of hypoglycemia, and consequently they needed some sweets.

*Then my four-years-old daughter said I need something sweet now… I have hypo… and she wiped her head. *(family member 12B).**

### 3.5. Watching Self-Management from the Sidelines

Taking care of diabetes is the responsibility of the person with diabetes. The family members watched their self-management from the sidelines. They told they saw many insulin injections and blood glucose self-measurements during the day. In some cases, they experienced some of those things unpleasant and did not want to see them. In other cases, the persons with diabetes followed the regimens so invisibly that the family members did not notice them at all. The only things they saw were different treatment equipment all over the home. The family members admired the persons with diabetes for the way they handled the illness.

*My wife injects herself next to the dining table. Not a pretty sight. *(family member 17B)**

*I can rely on my wife to handle diabetes… *(family member 10B).**

The family members also regarded themselves external in the management of diabetes. They saw how the persons with diabetes acted in everyday life but did not participate in any way in the diabetes regimen. They experienced themselves as outsiders. They avoided asking or commenting on any way and tried to stay in the background. By not asking anything, they wanted to respect the integrity of the persons with diabetes. They did not either want to be in the role of the controller. The family members also underrated their own role in the treatment of diabetes.

### 3.6. Living on an Emotional Roller-Coaster

The family members' feelings varied every day. Some days were full of worries and fears; some days were full of peace and confidence. To be afraid that something bad takes place was common among the family members. They felt different kinds of fears connected to both the present and the future. The fear of hypoglycemia was the most described. The fear of long-term complications was common as well, and family members even feared that the person with diabetes may die. Some of the fears were connected with self-management behaviour; for instance, the family members were afraid that the person with diabetes might forget to take insulin. They were also afraid that the equipment did not work, especially if the persons with diabetes used insulin pump therapy. The family members experienced uncertainty and confusion as well. They were unsure of how to act in accidental situations, and they were not confident in their abilities to help the persons with diabetes. They were not sure how the mood of the person with diabetes should be interpreted—whether it was caused by the level of blood glucose or something else. In spite of continuously attending to the condition of the persons with diabetes, the family members also experienced a sense of security. They trusted the future and did not fear long-term complications of diabetes. They trusted that by following the recommendations, the threats posed by diabetes could be avoided. They are still constantly attentive to the condition of the person with diabetes.

*The fear that something awful is going to happen is always on my mind. *(family member 2B)**

*I am not sure if I can help or if I should call for help or what I should do… *(family member 3B)**

*I can trust that mother wakes up, unless she has hypoglycemia during her sleep. *(family member 1B).**

## 4. Discussion

### 4.1. Study Limitations

In grounded theory research, there are usually many alternative sources of data. It is possible for example, to use interviews, observations, videos, drawings, and different kinds of written documents like diaries [30, 31]. In this study, only interviews were conducted and the family members were interviewed only once. By interviewing it is possible to get deeper information from the informants' point of view. It is also possible to ask specified questions during interviews. Keeping diaries would have been time consuming for family members and the observation of the family's everyday life would have been challenging to carry out and stressful to the family. Moreover, in this study, the family members had several-year experience of living with the people with diabetes and the data from interviews was rich and diverse. In the main part of the interviews, both the person with diabetes and the family member were present at the same time. This was the decision of the family or spouses and interviewer wanted to respect it [32]. It might have slightly affected how openly the family members talked about their feelings and thoughts. However, interviewees were in family's natural environment [33] and the atmosphere during interview was positive and relieved and in addition, also interactive discussing between spouses. The interviewer paid special attention to the atmosphere and convenience of the interview situation by encouraging the interviewees to talk freely and use their own words. Thus, although there were some preliminary questions for the interviews, the interviewer tried to keep the agenda and her mind as open as possible.

The data collection and analysis happen simultaneously in grounded theory research [30]. In this study, the themes in the interviews were based on what was discovered during the analysis of the previous interviews, which is according to grounded theory method [30, 34]. The data collection continued until no new properties and dimensions related to the concepts were found. The data were analysed using constant comparative analysis. Memo writing, which is important element of the analysis in grounded theory [30], was used to support the analysis. According to the grounded theory method, the last phase of the analysis is selective coding with the aim of identifying the core category and its links with the others [30]. In this study, it was very important to bring out the concepts describing the family members' point of view separately before combining the two datasets. After combining the two datasets, it was impossible to address the family members' point of view as clearly as it was before. In developing family-centred care and supporting especially the family members of adult persons with diabetes, it is important to know how they describe the everyday living with diabetes.

During a research process, it is important that the researcher is aware of his/her biases and assumptions [30]. The first author (the primary researcher and the interviewer) has worked as a diabetes specialist nurse, and her professional experience of diabetes may have influenced her communication with the informants, for example, by causing the use of very professional language. On the other hand, this experience might have been useful by enabling her to be more sensitive with the participants and the topic [30]. For example, diabetes-related slang words used by the family members in the interviews were already familiar to the interviewer. In this study, the primary researcher was aware of her preunderstanding of living with diabetes and wrote memos during the data collection and analysis [30, 34]. Regular meetings with the coauthors during the data collection and analysis were carried out to ensure the credibility of the categorisation.

Rigour was ensured by using several strategies: the selection of the participants was specified; during the interviews, the participants' concepts were used to develop new questions for the next interview; and the first author acknowledged possible preconceptions on the topic caused by previous professional experience. In addition, while presenting the findings, excerpts from the data have been used to support the findings [35, 36].

### 4.2. Discussion of Results

Everyday life with diabetes includes many concerns connected with diabetes. The risk of hypoglycemia is continuously on the family members' minds. They experience the fear of hypoglycemia, try to avoid it in every way, are prepared for it, and recognise it based on very mild signs. The family members are also afraid that something bad is going to happen or the person with diabetes develops long-term complications. Earlier studies [15, 21, 23] have also revealed that family members experience different kinds of fears and even more than the persons with diabetes themselves. Family members also consider hypoglycemia more serious than the person with diabetes [15].

Adherence to the self-management is a long process for the person with diabetes and for the family members. There are many barriers to optimal diabetes self-management faced by the person with diabetes [37, 38]. Self-management is affected by several factors, such as feelings, attitudes, self-efficacy [39, 40], knowledge and skills, and the motivation of the person with diabetes [41]. In addition, the support from the family members is important. In this study family members want to be involved in the management of diabetes. Similar results have been found by Stödberg et al. [21]. For support the person with diabetic family members needs knowledge and education [27]. In this study, the family members experience lack of knowledge. Still, they have not been offered the opportunity to participate in the education. For them, the only educator connected with diabetes is the person suffering from it. In their review Armour et al. [9] found that interventions including family members may be effective in improving knowledge and glycemic control. However, there is a little information about what kind of approaches are effective in family interventions.

There are other challenges the family members face as well. The family members experience uncertainty. In addition, the scheduled life with careful planning is not always experienced positively. Diabetes seems to influence everything and is always present. It must always be taken into consideration and it is impossible to do something spontaneously. Even the children of the family play games connected with diabetes. Similar findings conveying a picture of a restricted life with diabetes have also been reported earlier [24, 26]. Less information exists about the experiences of children with parents living with diabetes.

Self-management plays a crucial role in the management of diabetes; the person with diabetes is in charge. Nevertheless, family members participate in the daily self-management in many ways. They provide practical treatment connected to insulin therapy and blood glucose testing, they share the meals and exercise with the person with diabetes, and they discuss blood glucose levels and insulin titration with the person suffering from diabetes. On the other hand, some family members regard themselves as external; they are only bystanders of many self-management routines. They also underrate their role as a supporter of the person with diabetes. There is little earlier information about the family members' practical participation in the self-management of diabetes. Further research is needed on the expectations of their role in self-management.

This study provides new information about how family members of adult people with type 1 diabetes experience their everyday life. When comparing findings with earlier studies connected to the family members of children or adolescents [17–19], there are some similarities and some differences. Regardless of the age of the person with diabetes, family members have similar fears and worries. For example, the fear of hypoglycemia is described by adult family members as well. Similar findings connected with worries about hypoglycemia were found by Burns et al. [42].

Experiences of everyday living are individual and varying among the family members. There are many thoughts and feelings connected with diabetes, and mainly those feelings are negative. When supporting family members, it should be taken into consideration that negative feelings of family members might be barriers to their effective involvement in self-management [42].

When planning and implementing education programmes for adult patients with diabetes, spouses or family members should be encouraged to participate together with their partner or family member with diabetes. Developing family-centred interventions for adult people with type 1 diabetes and for their family members is needed (e.g., weekend or evening classes to couples or on-line chat rooms for spouses/family members). It is important that opportunities are provided for sharing thoughts and feelings concerning diabetes and self-management [43]. For example, sharing thoughts pertaining to hypoglycemia is paramount for reaching a good metabolic balance, because the fear of hypoglycemia may prevent it [44–46]. Outcomes of diabetes treatment are related to several factors and it is a challenge to study the influence of family on the outcomes. Interventions are needed that focus on the supportive aspects of family [29].

## 5. Conclusion

The findings of this study provide diverse information about the everyday life of families affected by diabetes and how the family members of adult people with diabetes experience their life. It is important to remember that adult people with type 1 diabetes do not live in a vacuum. Many of them have family members who are important to take into consideration in the wholeness of the diabetes management. These findings are valuable for developing education and for finding the best approaches to support adult people with diabetes and their families in meeting the challenges of living with diabetes. Further research is needed from the point of view of people with type 1 diabetes and their expectations towards family members. In addition, children's experiences connected with their parents' diabetes would be an interesting thing to find out.

## Figures and Tables

**Table 1:** An example of open coding.

Interview B	Substantive codes	Subcategory
“I said to my wife, you should make blood glucose measurement, I suppose it is low now”	Urging to perform blood glucose measurement	Helping during hypoglycemia
“She was little angry, so I said, you have to eat immediately”	Urging to eat	
“I feed jelly to my wife one night she could not eat by herself”	Feeding the person with diabetes	

**Table 2:** An example of axial coding.

Subcategory	Main category
Describing symptoms of hypoglycemia Recognising hypoglycemia Preparing for hypoglycemia Helping during hypoglycemia Avoiding hypoglycemia	Intertwining with hypoglycemia

**Table 3:** Background information of family members.

Gender	
Female	4
Male	15
Age	
Under 30	3
30–40	6
41–50	5
51–60	2
Over 60	3
Relationship with person with diabetes	
Wife	1
Husband	15
Daughter	3
Children in family	
No children	7
One child	2
Two children	3
Three children	1
Four children	1
Adult children	5
